# Working memory differences in long-distance dependency resolution

**DOI:** 10.3389/fpsyg.2015.00312

**Published:** 2015-03-23

**Authors:** Bruno Nicenboim, Shravan Vasishth, Carolina Gattei, Mariano Sigman, Reinhold Kliegl

**Affiliations:** ^1^Department of Linguistics, University of PotsdamPotsdam, Germany; ^2^Grupo de Lingüística y Neurobiología Experimental del Lenguaje, Instituto de Ciencias Humanas, Sociales y Ambientales, Consejo Nacional de Investigaciones Científicas y TécnicasMendoza, Argentina; ^3^Departamento de Física, Facultad de Ciencias Exactas y Naturales, Universidad de Buenos Aires/Instituto de Física de Buenos Aires, Consejo Nacional de Investigaciones Científicas y TécnicasBuenos Aires, Argentina; ^4^Escuela de Negocios, Universidad Torcuato Di TellaBuenos Aires, Argentina; ^5^Department of Psychology, University of PotsdamPotsdam, Germany

**Keywords:** locality, antilocality, working memory capacity, individual differences, Spanish, activation, DLT, expectation

## Abstract

There is a wealth of evidence showing that increasing the distance between an argument and its head leads to more processing effort, namely, locality effects; these are usually associated with constraints in working memory (DLT: Gibson, 2000; activation-based model: Lewis and Vasishth, 2005). In SOV languages, however, the opposite effect has been found: antilocality (see discussion in Levy et al., 2013). Antilocality effects can be explained by the expectation-based approach as proposed by Levy (2008) or by the activation-based model of sentence processing as proposed by Lewis and Vasishth (2005). We report an eye-tracking and a self-paced reading study with sentences in Spanish together with measures of individual differences to examine the distinction between expectation- and memory-based accounts, and within memory-based accounts the further distinction between DLT and the activation-based model. The experiments show that (i) antilocality effects as predicted by the expectation account appear only for high-capacity readers; (ii) increasing dependency length by interposing material that modifies the head of the dependency (the verb) produces stronger facilitation than increasing dependency length with material that does not modify the head; this is in agreement with the activation-based model but not with the expectation account; and (iii) a possible outcome of memory load on low-capacity readers is the increase in regressive saccades (locality effects as predicted by memory-based accounts) or, surprisingly, a speedup in the self-paced reading task; the latter consistent with good-enough parsing (Ferreira et al., 2002). In sum, the study suggests that individual differences in working memory capacity play a role in dependency resolution, and that some of the aspects of dependency resolution can be best explained with the activation-based model together with a prediction component.

## 1. Introduction

Long-distance dependencies (also called non-local, filler-gap, or unbounded dependencies) have been investigated since Fodor's (1978) work on parsing strategies, but many questions remain unanswered or only partially answered. It is uncontroversial that the distance over which a dependency is resolved, shown in (1) with an arrow, is a primary determinant of the speed and the accuracy of the dependency resolution (among others: Gibson, 2000; McElree et al., 2003; Lewis and Vasishth, 2005; Levy, 2008). It is controversial, however, how increasing this distance affects the speed and the accuracy of the resolution.


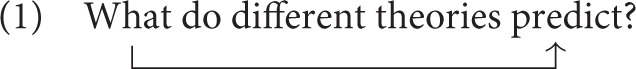


### 1.1. Memory-based explanations

There is a wealth of evidence showing that increasing the distance between an argument and its head hinders underlying memory processes in some way. This is supported by research that shows that longer dependencies produced (i) locality effects, that is, a slowdown (or increase of regressive saccades) at the region of the dependency resolution when the distance between dependent and head or subcategorizing verb (or gap) is increased (either in self-paced reading, eye-tracking experiments, or both; among others: Gibson, 2000; Grodner and Gibson, 2005; Demberg and Keller, 2008; Bartek et al., 2011; Vasishth and Drenhaus, 2011); (ii) Event Related Potential (ERP) measures associated with difficulty (Kluender and Kutas, 1993; Fiebach et al., 2002; but see: Phillips et al., 2005); and (iii) deterioration of response accuracy in speed-accuracy trade-off (SAT) experiments (McElree, 2000; McElree et al., 2003). The underlying memory process that is adversely affected when distance is increased is subject to debate. Here we discuss two theories that account for the memory-based locality effects: dependency locality theory (DLT; Gibson, 2000) and the activation-based model (Lewis and Vasishth, 2005).

DLT posits two separate components of a sentence's processing cost: storage and integration costs. Storage cost is argued to depend on the number of syntactic heads required to complete the current input as a grammatical sentence (Gibson, 2000) and seems to be independent of the amount of time that an incomplete dependency is held in memory (Gibson et al., 2005). On the other hand, integration cost is locality-based, that is, the cost is based on the distance between the dependent and its head; this distance is based on the number of new intervening discourse referents (Gibson, 2000).

In contrast to DLT, which is a theory specific to sentence comprehension processes, the activation-based model is based on a general cognitive model. In the activation-based model, linguistic items in memory are represented as feature bundles that suffer from decay and interference from the features of other linguistic items. Under this model, locality effects can be explained in terms of difficulty in the retrieval of a non-local argument; retrieval is driven by cues that are set at the moment of dependency resolution. Since the access to the argument involves a match of retrieval cue features against candidate memory items (Lewis et al., 2006), this access is adversely affected when (i) more time has passed from the encoding of the argument (decay); and (ii) when there are other items with similar features that serve as distractors (similarity-based interference). The activation-based model excludes the possibility of storage costs as proposed by DLT, but stored memories have their observable effects through interference (Van Dyke and Lewis, 2003; Lewis et al., 2006).

Thus, in cases such as (2), both DLT and the activation-based model predict that as the distance between the displaced argument *who* and the subcategorizing verb *supervised* increases, the retrieval of the argument will be harder. This is supported by the evidence of locality effects in relative clauses (Grodner and Gibson, 2005; Bartek et al., 2011).

(2)     From Experiment 2 of Grodner and Gibson (2005)
          a.  The administrator **who** the nurse **supervised**…
          b.  The administrator **who** the nurse from the clinic **supervised**…
          c.  The administrator **who** the nurse who was from the clinic **supervised**…

In spite of the evidence for locality effects, there is a growing body of evidence showing the opposite effect: antilocality. Studies on SOV structures (in Hindi: Vasishth, 2003; Vasishth and Lewis, 2006; and in German: Konieczny, 2000; Konieczny and Döring, 2003; Levy and Keller, 2013) showed that increasing distance can produce a speedup at the site of the dependency completion. In many cases the speedup can be accommodated in the activation-based model since the interposed material can help to strengthen the representation of the upcoming head by activating it through modification (Vasishth and Lewis, 2006). This would entail that the processing of the head will be facilitated since it has already been generated; we will express that here by saying that the VP has been *preactivated*. This is specially relevant for SOV languages, where the arguments of the VP appear preverbally, modifying the VP before the head is parsed. So, in cases such as (3), where the extra material belongs to the VP, the activation-based account will predict that increasing distance should, in fact, result in a speedup (but only if the decay does not offset the benefit of activation; Lewis et al., 2006).

(3)     From Vasishth and Lewis (2006)
          a.  Vo  kaagaz **jisko**  us   laṛke-ne   **dekhaa** bahut
               that paper  **which** that boy-ERG  **saw**       very
               puraanaa thaa.
               old          was
               ‘That paper which that boy saw was very old.’ (Object relative, no intervening discourse referents)
          b.  Vo  kaagaz **jisko**  us   laṛke-ne   mez-ke          piiche
               that paper  **which** that boy-ERG  table-GEN   behind
               gire.hue **dekhaa** bahut puraanaa thaa.
               fallen     **saw**       very  old           was
               ‘That paper which that boy saw fallen behind a/the table was very old.’ (Object relative, two intervening discourse referents)

### 1.2. Expectation-based explanations

As in other aspects of cognition, predictions play an important role in language, and evidence from different sources supports the view that language processing does not only depend on bottom-up processes (for a review of prediction in language see: Kutas et al., 2011). It has been shown that a syntactically constraining context can lead to facilitation when a word is predicted either (i) because of local syntactic constraints related to characteristic of verbs, as proposed by Trueswell et al. (1993), and Konieczny (2000); or (ii) because the parser is able to build structure in a top-down manner, using grammatical or probabilistic information, as proposed by Jurafsky (1996) and Hale (2001). The latter idea was developed further in an expectation-based theory of processing (Levy, 2008) where the main source of difficulty is determined by the surprisal (negative log of the conditional probability) of a word given its context (as proposed by Hale, 2001). The surprisal metric proposed by Hale (2001) formalizes the idea that a more surprising lexical content is also less predictable.

Long-distance dependency resolution is a situation where the comprehender knows that a subcategorizing verb has to appear, but does not know exactly when. Since each constituent of a given category that is integrated after the dependent (a wh-element in this case) eliminates most of the expectation for seeing another constituent of the same type next, each constituent that is read increases the expectation for seeing a constituent of one of the remaining types. Because the subcategorizing verb is one of the remaining types, the expectations of finding it will increase monotonically, and being more expected it will also be processed more easily. In other words, given that the clause has a finite length, the probability that the next word will be the subcategorizing verb rises as the number of words after finding the wh-element increases (in a similar way to an increasing hazard function as proposed for visual search by Peterson et al., 2001, and for the anticipation function of environmental cues in macaques by Janssen and Shadlen, 2005).

Thus, also in the cases where memory-based accounts will predict locality effects (due to integration or retrieval costs), the expectation-based account of dependency resolution will predict the opposite effect: antilocality. The predictions of the expectation-based account for non-local dependency resolution were borne out specially in studies using languages with SOV structures, which showed antilocality effects. However, as mentioned before, in many cases the predicted antilocality effects could also be explained either with local syntactic constraints (Konieczny, 2000) or with an activation-based account (Vasishth, 2003; Vasishth and Lewis, 2006). Independent support for the expectation-based account of antilocality in dependency resolution would come from cases where the length manipulation is independent of material that belongs to the VP and appears preverbally. Cases like this can be found in length manipulations such as (4): object wh-questions where the dependency crosses over a sentence boundary. This is examined in more depth in the experiments of this paper.

(4)     a.  **Who** has John **called**?
          b.  **Who** does Mary think that John has **called**?

### 1.3. Individual differences

#### 1.3.1. Working memory capacity and the parsing of unbounded dependencies

Memory-based accounts of locality effects assume, either implicitly or explicitly, that if more working memory capacity (WMC) is required for processing than is available, longer processing times and/or a higher proportion of errors will result during retrieval or integration. This prediction is implicit in DLT, where the upper limits on storage and integration cost (Gibson and Thomas, 1999; Gibson, 2000) should depend on WMC; and it is explicit in the activation-based model, where low capacity is argued to result in hindered ability to complete a retrieval (Daily et al., 2001). One plausible implication is that low-capacity readers may be more affected by locality effects, showing stronger effects than high-capacity readers.

However, the effect of individual differences in WMC influencing dependency resolution processes has been neglected in the literature (but see: Van Dyke et al., 2014). This absence of work is surprising given that there is considerable evidence for the interaction of individual differences with syntactic and semantic processes (Just and Carpenter, 1992; Pearlmutter and MacDonald, 1995; Traxler et al., 2005, 2012; von der Malsburg and Vasishth, 2013), and there is also evidence for a reduction in performance during long-distance dependency resolution and memory dual-tasks (Fedorenko et al., 2006, 2013).

Regarding the influence of working memory on expectation-based parsing, the predictions are less clear. The studies showing that expectations may play a dominant role only when working memory load is relatively low (Levy, 2008; Levy and Keller, 2013; Husain et al., 2014) suggest that the processes involved in the anticipation of upcoming material may also depend on working memory. This is so because comprehenders' expectations depend on the accumulating information (Levy, 2008). Low-WMC readers, who have a reduced ability to temporarily store and manipulate information, may then be less able to adequately expect upcoming lexical material, relative to high-WMC readers. To our knowledge, the only evidence for this claim, however, comes from Otten and Van Berkum's (2009) ERP study where low-WMC participants showed an additional later negativity (900–1500 ms) to unexpected content.

#### 1.3.2. WMC and reading skills

Differences in WMC can successfully explain individual differences in comprehension performance (Daneman and Carpenter, 1980); and this measure of individual differences seems to be the right candidate to account for differential effects in processes related to dependency resolution. There is ample evidence showing that lower WMC reflects higher limitations in attention allocation for goals (Engle, 2002), and several studies have shown the predictive power of WMC for language comprehension ability (for a meta-analysis of 77 studies till the mid-nineties: Daneman and Merikle, 1996). Furthermore, some studies have shown that individuals with lower capacity are less successful in integrating information over distance in a text (Daneman and Carpenter, 1980; Yuill et al., 1989), and have greater comprehension deficits, in part, because they are less able to maintain on-task thought (McVay and Kane, 2011). Moreover, low-capacity participants seem to have a greater disadvantage than high-capacity participants when they face difficult sentences (for garden-path vs. non-garden path sentences: Christianson et al., 2006; for comprehension reaction times in subject- vs. object-relative clauses: King and Just, 1991; Vos et al., 2001). The reason for differences in WMC may be rooted in the variability in either a limited amount of activation (Just and Carpenter, 1992; van Rij et al., 2013), computational resources available or processing efficiency (among others: Daneman and Carpenter, 1980; Daneman and Carpenter, 1983), the ability to overcome interference (Hasher and Zacks, 1988; Unsworth and Engle, 2007), or the efficiency of retrieval cues present in the active portion of working memory (Ericsson and Kintsch, 1995).

It is possible, however, that individual differences in capacity only reflect experience and not intrinsic capacity differences (MacDonald and Christiansen, 2002; Wells et al., 2009). Readers characterized as high-capacity may indeed be more sensitive to the semantic cues available to them, as proposed by Pearlmutter and MacDonald (1995), but mainly because these readers also have more language experience. In fact, recent work by Traxler et al. (2012) raises the concern that WMC correlates with many other reader characteristics. According to Traxler et al., fast readers, who read more often than slow readers, will have greater experience with language; this would in turn make them more sensitive to semantic cues in the syntactic analysis. In a new set of analyses based on Traxler et al.'s (2005) data set, Traxler and colleagues found that WMC interacted with sentence-characteristic variables only when reading speed was not included in the model (since they assumed that reading speed was a measure of reading skills).

In order to obtain a reliable measure of working memory that is not correlated with reading speed and experience, we chose to use the operation span task (Turner and Engle, 1989; Conway et al., 2005). In addition, we adopted the rapid automatized naming task (Denckla and Rudel, 1976), since it has been shown that it predicts reading speed, comprehension, and other characteristics associated with reading skills (among others: Kuperman and Van Dyke, 2011). The inclusion of both tasks can therefore help to determine whether it is WMC and/or reading experience that account for differences in dependency resolution processes.

## 2. Experiments

The experiments have two main objectives. The first objective is to disentangle memory- and expectation-based explanations on the processing of long-distance dependencies. While both the expectation and activation accounts may predict antilocality effects, the activation-based model predicts that facilitation should occur when intervening material modifies an upcoming head, whereas the expectation account predicts facilitation regardless of what the intervening material modifies. Even though this is an oversimplification of the expectation account as defined by Hale (2001) and Levy (2008), it should hold for the type of sentences we included in our stimuli.

The second objective is to examine the effect of individual differences in dependency resolution: if working memory constraints are involved, participants with different WMC should show differential locality or antilocality effects.

In order to address these objectives, we measured WMC and reading skills of (Argentinean) Spanish native speakers, and we used both self-paced reading and eyetracking methodologies to provide converging evidence. The use of Spanish stimuli allowed us to investigate antilocality effects in an SVO language. In addition, because of the relatively free order and long sentences permitted by Spanish, we could do a manipulation that is more common in studies that investigate antilocality in *SOV* structures: increasing the dependent-head distance by interposing material that belongs to the verbal phrase (VP) but appears prior to the verb.

The design of the stimuli is exemplified by (5). The distance between the wh-element and the head verb (*had fired*) was manipulated by including an adverbial phrase (AdvP; *before some days*) that attaches to the different VPs in the sentence. Hence there are two different aspects of the manipulation to consider for each condition: (i) the attachment site of the adverbial phrase (main VP, intermediate VP, and last VP where the dependency is completed) and (ii) the length of the dependency between the wh-word (who.ACC) and the head verb. In (5a), the length of the dependency is the shortest one, since the AdvP is attached to the main clause VP *asked* (henceforth condition VP1). This entails that by the time the dependency is started at the wh-element, the AdvP has already been interpreted. In this condition, the action that was performed *before some days* was the “asking.” In both conditions (5b) and (5c) the dependency length is larger than in (5a), since the the AdvP is interposed between the dependent and head verb. However, while in (5b) the AdvP modifies an intermediate VP (henceforth condition VP2), in (5c) it modifies the third VP, which contains the head verb, where the dependency is completed (henceforth condition VP3). So while in condition VP2 the “saying” happened *before some days*, in condition VP3 the “firing” of the dependent “who.ACC” was *before some days*. All the items had as a second verb either *comentar* or *decir* “to say.” Even though these two verbs are ditransitive, the ditransitive construction is extremely uncommon in Argentinean Spanish without a clitic. This means that the reading that would allow an indirect object such as *a quién* completing the dependency is very unlikely (for a similar construction in Spanish with clitic left-dislocation, see Pablos, 2006). Since this type of verbs appears in all conditions, and they are not in the region of interest, they should not affect the experiment. Notice, as well, that the head verb position is kept fixed across conditions in order to avoid word-position effects (Ferreira and Henderson, 1993). The characteristics of the stimuli are summarized in Table 1.

(5)     a.  attachment at VP1
              Hace algunos días, José preguntó **a quién**
              Before some days  José asked     **who.ACC**
              comentaron   que  el   gerente      **había despedido**
              they-said       that  the manager    **had fired**
              por             equivocación.
              because-of mistake
              “Some days ago, José asked who they said that the manager had fired by mistake.”
          b.  attachment at VP2
              José preguntó **a quién**,   hace algunos días,
              José asked     **who.ACC** before some days
              comentaron que el   gerente   **había despedido**
              they-said     that the manager **had fired**
              por             equivocación.
              because-of mistake
              “José asked who they said some days ago that the manager had fired by mistake.”
          c.  attachment at VP3
              José preguntó **a quién**    comentaron que,
              José asked     **who.ACC** they-said     that
              hace algunos días, el   gerente   **había despedido**
              before some days  the manager **had fired**
              por             equivocación.
              because-of mistake
              “José asked who they said that the manager had fired some days ago by mistake.”

**Table 1:** **Summary of the conditions**.

**Cond**.	**Constituent modified by AdvP**	**Dependency length**
VP1	Main VP (head: asked)	Short
VP2	Intermediate VP (head: said)	Long
VP3	VP where the dep. is completed (head: had fired)	Long

### 2.1. Predictions

Predictions for the critical region (head verb) are summarized in Table 2. When the dependency length is increased (VP2 vs. VP1 and VP3 vs. VP1), DLT predicts increased processing effort, that is, locality-effects. In contrast, the expectation account predicts facilitation at the head verb, that is, antilocality effects. The activation-based model predicts, similar to DLT, increased processing effort for both VP2 and VP3 due to the decay of the wh-element. However, in contrast to DLT, the activation-based model also predicts that in VP3 this difficulty should be counteracted by the preactivation of the VP that contains the head verb. According to the activation account, while VP2 should display locality effects, the effect displayed by VP3 should depend on which underlying process is stronger: activation or decay (which in turn should depend on WMC).

**Table 2:** **Summary of the conditions and predictions for the head of the dependency**.

**Cond**.	**Expectation account**	**Memory-based accounts**	
		**DLT**	**Activation**
VP1	Baseline	Baseline	Baseline
VP2	Facilitation	Difficulty	Difficulty
VP3	Facilitation	Difficulty	Difficulty and Facilitation

It should be noted that while for self-paced reading experiments stronger locality effects imply longer reading times (Gibson, 2000; Grodner and Gibson, 2005; Bartek et al., 2011) and stronger antilocality effects imply shorter ones (Konieczny, 2000; Vasishth and Lewis, 2006; Levy, 2008), for eye-tracking studies these effects have been associated with different measures. Locality has been associated with the increase in the duration of first pass reading times in Staub (2010), total reading times and second pass reading in Bartek et al. (2011) and Levy and Keller (2013), and higher re-reading probabilities in Vasishth and Drenhaus (2011); and antilocality with the reduction of the duration of total reading times and second pass reading in Levy and Keller (2013), regression-path durations in Konieczny and Döring (2003), and lower first-pass regression probabilities in Vasishth and Drenhaus (2011).

**Figure 1:**
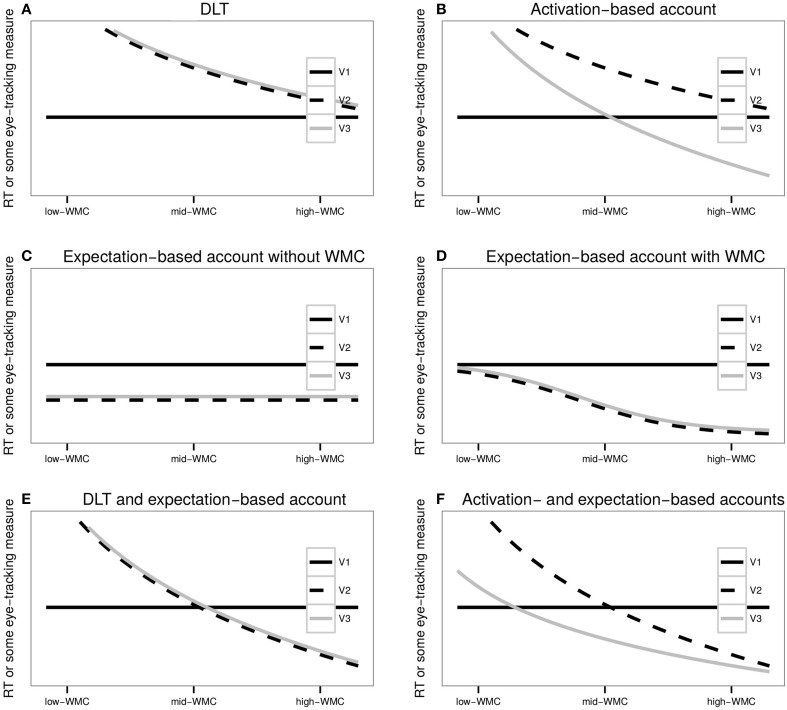
**The figure depicts the predictions of (A) DLT, (B) activation-based account, (C) expectation-based account unaffected by WMC, (D) expectation-based account affected by WMC, (E) the combination of the predictions of DLT and the expectation-based account, and (F) the combination of the predictions of the activation- and the expectation-based accounts**.

Since the processing efforts of DLT and the activation account are associated with working memory constraints, according to these memory-based theories, participants with different WMC should show differential effects: the parse of the critical region will require more processing effort for low-WMC readers than for high-WMC. Thus, DLT predicts that as WMC increases, locality effects should decrease; and for high WMC (compared to low WMC) there should be the smallest difference between long and short conditions (see Figure 1A). For the expectation account, it is not clear whether WMC plays a role at all. If WMC is not relevant, there should not be a differential effect depending on the WMC of the readers (as in Figure 1C). It may be the case, however, that readers with more WMC are able to predict upcoming material better, then they should also display stronger antilocality effects (till a certain limit: either a minimal duration of the fixations or reading times or virtually no re-reading, as it is seen in Figure 1D). Regarding the activation-based account, its prediction for condition VP2 should be the same as the one of DLT: as WMC increases, locality effects should decrease; however, for condition VP3 the locality effects should be counteracted with facilitation due to preactivation, and given enough WMC, readers should offset the processing efforts and display antilocality effects (Figure 1B).

However, expectation and memory-based theories are not mutually exclusive; recent research supports the idea that insights from both types of theories are needed (Staub, 2010; Vasishth and Drenhaus, 2011; Levy and Keller, 2013; Levy et al., 2013; Husain et al., 2014). If DLT acts together with the expectation account (either the type that does not depend on memory, see Figure 1C, or the one that does depend on memory, see Figure 1D), locality effects should decrease as WMC increases until they become increasing antilocality effects, but, as before, the facilitation should not exceed a certain lower limit (see Figure 1E). As it is the case with each of these two accounts independently, the combination of DLT with the expectation account does not predict any difference between VP2 and VP3. If the activation-based model acts together with the expectation account, locality effects should also decrease together with an increase of WMC till they become increasing antilocality effects. However, processing efforts should be weaker and facilitation stronger for VP3 in contrast to VP2, since the facilitation of VP3 has two sources: expectations and preactivation, while the source of facilitation in VP2 is only expectations (see Figure 1F).

### 2.2. General procedure

Participants were tested individually using a PC computer. They got an overview of the whole experiment and then completed three tasks at their own pace: First, they performed a rapid automatized naming task; second, an operation span task; and finally, subjects performed an eye-tracking experiment in Experiment 1, and a self-paced reading task in Experiment 2.

#### 2.2.1. Operation span task

Participants took part in the operation span task (Turner and Engle, 1989) using a software developed by von der Malsburg (https://github.com/tmalsburg/py-span-task) and used in von der Malsburg and Vasishth (2013) following the recommendations given in Conway et al. (2005). Even though variants of the reading span task by Daneman and Carpenter (1980) have been used in many psycholinguistic studies, it is likely that the reading span task measures verbal ability or reading experience as well as WMC (MacDonald and Christiansen, 2002; Conway et al., 2005). Since reading experience is also a good candidate for explaining differential effects in sentence processing, a solution is to include a nonverbal task to examine the domain-general aspects of cognition that may contribute to the individual differences (Swets et al., 2007). Since the operation span task probably measures mathematical ability as well as working memory (but not reading skills), if higher scores of the operation span task predict facilitation between experimental conditions, it would be unlikely that the result could be explained by the effect of reading experience alone.

The procedure of the operation span task test was similar to the one employed by von der Malsburg and Vasishth (2013) with some minor modifications: First, participants had to verify the correctness of 25 simple equations. At this stage, the reaction time of the Equations 10 to 25 was measured; the average reaction time plus two standard deviations was used as a time-out at the second stage. Calculating a time-out for every participant ensures that participants that are fast will not have time left to rehearse the items at the following stage of the test. Afterwards, participants had to carry out a dual task: check equations and memorize letters that were shown between the equations for 800 ms. After a group of equation-letter successions, participants were instructed to type in order the letters that had appeared before.

Before participating in the actual test, subjects practised with four trials of equation-letter successions. In the main test, successions of equation-letter had between three and seven elements, and there were eight sets for each size resulting in 32 trials. Presentation order of the sets was randomized and no feedback regarding the correctness of the judgments of equations or recalled items was given.

In all parts of the test, participants had to read the equations and letters aloud in order to prevent vocal rehearsal strategies. Only consonants were used as memory items to prevent participants from forming “words” with vowels and consonants, or “sentences,” if words had been used.

Partial-credit unit scores, which indicate the mean proportion of correctly recalled items within the sets (Conway et al., 2005), were used as a numeric score of individual working memory.

#### 2.2.2. Rapid automatized naming tasks

Working memory-capacity correlates with other reader characteristics, which may in turn account for the variance in participants' reading behavior as well as or better than working memory capacity (Traxler et al., 2012). To determine whether working memory capacity correlates with reading times independently of reading skills, it is important to assess the effects of working memory capacity in the presence of some measure of reading skills.

Even though there are different ways to measure reading skills (among others: speeded naming abilities, oral language ability, vocabulary, attention), Kuperman and Van Dyke (2011) analyzed which tests from a broad battery of individual difference measures were predictive of eye-movement patterns associated with reading ability. They showed that rapid automatized naming was a robust predictor across the entire eye-movement record.

Participants with longer rapid automatized naming times tend to have lower reading comprehension scores, slower reading rates and their initial landing position when fixating tends to be further to the left (among others: Howe et al., 2006; Arnell et al., 2009; Kuperman and Van Dyke, 2011). Moreover, rapid automatized naming tasks seem to recruit a network of neural structures also involved in more complex reading tasks (Misra et al., 2004). In normal reading, readers must be able to disengage from one stimulus and move to another, rapidly programming saccades as the eyes move. Since this task involves speeded serial visual inspection and subsequent naming of items, the oculomotor component of this task is very similar to that required in natural reading.

In order to measure rapid automatized naming times, the first author developed a software that automatizes the test (https://github.com/bnicenboim/py-ran-task). In this task, participants saw a grid containing items (either letters or digits), and they were instructed to name them as fast as possible.

Each subject read a series of screens with 50 items; the items were the same set of letters or numbers that were used in Denckla and Rudel (1976): {o, a, s, d, p} and {2, 6, 9, 4, 7}. The first eight trials were composed of letters and the following eight had numbers. The items were displayed in five rows of ten columns and were listed in random order with the constraint that adjacent items were not the same. Before every trial, a screen with underscores instead of the items was displayed.

The participants were instructed to read aloud as fast as possible, and in case they misread, they were instructed to reread only the misread item. The test started with two practice trials to familiarize the participants with the task. Each trial started and ended with the spacebar: participants were instructed to start reading immediately after pressing the spacebar, and to press it again immediately after finishing reading aloud the last item.

Since the total reading times for letters and for numbers were highly correlated (*r* = 0.88 for Experiment 1 and *r* = 0.87 for Experiment 2), both were averaged together. The inverse of this averaged reading time was used as the *reading skills measure*; this way the measure furnishes an intuitive value associated with speed: a higher value represents a more skilled reader.

#### 2.2.3. Data analysis

The data analysis was conducted in the R programming environment (R Core Team, 2013), using either linear mixed-effects model (LMM; Pinheiro and Bates, 2000) or generalized linear mixed-effects models with a binomial link function to the response data (GLMM). Both are regression models that include both fixed effects (such as predictors) and random effects, and they are available in the package *lme4* (Bates et al., 2015). Since LMMs minimize the false positives when they include the maximal random effects structure justified by the design (Schielzeth and Forstmeier, 2009; Barr et al., 2013), both LMMs and GLMMs were fit following this guideline. However, the random effects structure was simplified by removing the correlations, since the models either did not converge or the correlation between variance components could not be estimated.

For large samples, the *t* distribution approximates the normal distribution and an absolute value of *t* larger than 2 indicates a significant effect at α = 0.05. For all the models presented in the study, covariates such as WMC and reading skills were scaled and centered.

## 3. Experiment 1

### 3.1. Method

#### 3.1.1. Participants

Seventy-six subjects aged between 17–42 years old (mean 24.1 years) participated in this experiment in Buenos Aires, Argentina. All participants were native speakers of Spanish and were naïve as to the purpose of the study. Five participants were excluded from the analysis: two participants had reading glasses that prevented an adequate calibration of the eye-tracker, two performed poorly in the mathematical task of the operation span test (with less than 70% accuracy), and another subject reported that she consciously re-read every sentence.

Partial-credit unit scores (Conway et al., 2005) for the operation span test measuring WMC of the remaining 71 participants ranged between 0.232–0.801 with an average of 0.543 (*SE*: 0.013). Average character speed for the rapid automatized naming task for measuring reading skills ranged between 1.44–3.72 characters/second with an average of 2.54 (*SE*: 0.06) characters/second.

#### 3.1.2. Stimuli

The stimuli for this experiment consisted of 48 items with three conditions (place of attachment) similar to example (5). Each participant read the 48 items together with 120 unrelated sentences (72 were experimental items of two unrelated experiments and 48 sentences were filler sentences) in an individually randomized order. The 144 experimental sentences (48 items in three conditions each) were presented in Latin square design. In order to ensure that participants had paid attention to the sentences, a true-or-false comprehension task was presented after half of all trials in the experiment, including fillers. Half of these statements were true and half false. For the sentences in (5), for example, the statement was false and it was the following: *El gerente fue despedido por equivocación*. “The manager was fired by mistake.” The statements following other experimental sentences focused on different aspects of the stimuli: the participants (such as “Jose fired someone.”), the action (“The manager hired someone.”), the setting of the action (such as “Someone was fired on purpose.”), etc. We chose to use true-or-false statements instead of yes-no questions in order to avoid long and unnatural questions.

#### 3.1.3. Procedure

Participants performed the eye-tracking task after having completed a rapid automatized naming task and an operation span task. Before the eye-tracking experiment began, each participant was instructed to read for comprehension in a normal manner and had a practice session of seven sentences. Eye movements were recorded using an EyeLink 1000 eye-tracker, interfaced with a PC. Stimuli were displayed on an 21” monitor. Subjects were seated 65 cm from the computer screen. Viewing was binocular, but only the right eye was recorded. All sentences were displayed on a single line and were presented in twelve points Arial font. At the beginning of each trial, a dot appeared at the left edge of the screen and after participants fixated on this dot, the sentence appeared. Participants had to look at the bottom right corner of the screen to indicate they had finished reading. True-or-false statements appeared randomly for half of the stimuli at this point. No feedback was given as to whether the response was correct or not. After reading half of the sentences, participants took a 10-min break. A calibration procedure was performed at the beginning of the eye-tracking experiment, at the end of the break, and between trials as needed.

#### 3.1.4. Data analysis

Detection of saccades and fixations was done using a modification of the saccades package developed by von der Malsburg (https://github.com/tmalsburg/saccades), and eye-tracking measures were computed using *em2* package (Logačev and Vasishth, 2013). The appropriate transformation of the dependent variable was determined using the Box-Cox method (Box and Cox, 1964; Kliegl et al., 2010) with the boxcox function in the MASS package (Venables and Ripley, 2002). The log transformation was suggested as the most appropriate transformation.

### 3.2. Results

#### 3.2.1. Comprehension accuracy

Participants answered correctly on average 80% (*SE*: 1) comprehension probes of all trials, and 82% (*SE*: 1) of the trials belonging to the experiment. The comprehension accuracy for the experimental trials ranged between 58 and 100%, while the 25th, 50th, and 75th quartiles were 75, 83, and 90% respectively. In addition, a GLMM showed that WMC was a significant predictor of accuracy (higher capacity led to greater accuracy); Coef = 0.21, *SE* = 0.10, *z* = 1.98, *p* = 0.048.

#### 3.2.2. Eye-tracking measures

Reading times were inspected at three regions of interest: the first critical region (auxiliary verb “había”), second critical region (participle form of the verb), and spillover region (P). We used successive differences contrast coding to test the predictions of the different accounts: VP2 (coded as 1) against VP1 (coded as −1) and VP3 (coded as 1) against VP2 (coded as −1). As in Vasishth and Drenhaus (2011), we found effects in the critical regions only in dependent measures related to re-reading; in the spillover region, we found effects only for total fixation time, consistent with Levy and Keller (2013). We provide the analysis of regions of interest for first-pass regression probability, re-reading probability and total fixation time. As defined in Vasishth and Drenhaus (2011), first-pass regression probability at a word is the probability of the eye moving leftwards after this word was fixated at least once; re-reading probability for a word is the probability of revisiting that word after having having made a first-pass.

After inspecting each LMM with total fixation time as dependent variable, we removed 0.12% of the data in order to keep the residuals normally distributed; the results of the model were virtually the same without this removal. Below we report only statistically significant effects.

##### 3.2.2.1. First critical region (auxiliary verb “había”)

**Figure 2:**
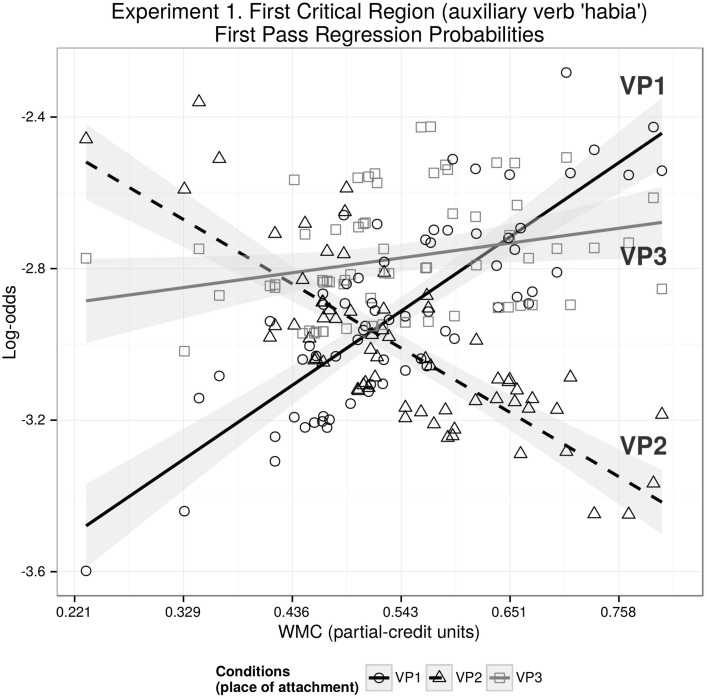
**The figure depicts the partial effects on first pass regression probabilities in log-odds scale for the contributing factors condition, WMC, and their interaction; random factors variance and effects due to reading skills were removed from the dependent variable using the remef function (Hohenstein and Kliegl, 2013)**.

We found a WMC and VP2-VP1 interaction for first-pass regression probabilities (Coef = −0.38, *SE* = 0.17, *z* = −2.17, *p* = 0.03) showing that as WMC increases, the probability of a regression at the auxiliary verb decreases for condition VP2 in comparison with VP1 (as shown in Figure 2).

Since we did not find evidence of more facilitation in VP3 in comparison with VP2, we also fitted a separate model that included the VP3-VP1 contrast. We found a decrease in re-reading probability for VP3 in comparison with VP1 (Coef = −0.28, *SE* = 0.12, *z* = −2.40, *p* = 0.016).

##### 3.2.2.2. Second critical region (participle form)

As in the first critical region, we found a decrease in re-reading probabilities for VP3 condition in comparison with VP1 (Coef = −0.20, *SE* = 0.10, *z* = −1.99, *p* = 0.047).

##### 3.2.2.3. Spillover (preposition)

We found a significant speedup for VP2 in comparison with VP1 for total reading time (Coef = −0.06, *SE* = 0.03, *t* = −2.07), and an unpredicted interaction between reading skills and VP2-VP1 (Coef = 0.09, *SE* = 0.03, *t* = 2.86) showing that as reading skills increases, total reading times at the spillover for condition VP2 increase in comparison with condition VP1.

### 3.3. Discussion

The central finding in the eye-tracking study is that individual differences associated with working memory have an impact in parsing sentences with long-distance dependencies. When the extra material modifies the intermediate VP (VP2), results for first pass regression probabilities for the critical region are consistent with the idea that expectations play a dominant role when the individual capacity of the participants is large enough to overcome the memory-driven locality effects (see Figure 2). That is, locality effects may become antilocality effects when WMC is large enough. This pattern can be explained by a memory account acting together with the expectation account. However, from this pattern alone it is not clear whether DLT or the activation-based model best explain the data. The predictions of DLT are based solely on dependency length, entailing that VP2 should be fully aligned with VP3 (see Figure 1E). The activation-based model predicts facilitation when the extra material is attached to the head verb, that is, facilitation for VP3 in comparison with VP2 (while sharing the same lower asymptote for extremely high WMC; see Figure 1F). At least for first pass regression probabilities for the critical region, it is unclear where VP3 condition stands: there is no significant facilitation in comparison with VP1 as all the described accounts would predict.

However, the study does provide some evidence for a differential effect that depends on *where* the extra material is attached, and not just on the linear distance of the dependency (as DLT and expectation account would predict). When the extra material is part of the same VP as the subcategorizing head verb (VP3), re-reading probabilities show facilitation compatible both with expectations and with the preactivation of the subcategorizing verb and similar to the evidence from SOV languages (Konieczny, 2000; Konieczny and Döring, 2003; Vasishth, 2003; Vasishth and Lewis, 2006; Levy and Keller, 2013). The fact that facilitation occurs only for VP3 condition in comparison with the short dependency condition VP1, and not when the extra material modifies the intermediate VP (VP2), provides some indirect evidence indicating differential facilitation between VP2 and VP3 as predicted by the activation account.

As mentioned before, one of the main differences between the predictions depicted in Figure 1 and our results is the status of VP3 condition: The facilitation of VP3 in comparison with the baseline VP1 appears in a different measure (re-reading instead of regression probabilities) than the facilitation of VP2 condition (in comparison with VP1), and it “spilled over” to the second critical region. In addition, and in contrast with VP2, the facilitation did not depend on the WMC of the participants.

Regarding the differences in the eye-tracking measures and spillover, the effect of adding preverbal material may have been more complex than hypothesized. The preverbal material may have added a new retrieval process at the head and thus overshadowed any facilitation caused by increased expectations. Furthermore, the appearance of the facilitation in different measures can be accounted for by assuming that facilitation due to preactivation, and facilitation due to increased expectations depend on different underlying mechanisms resulting in qualitatively different behavioral consequences in reading (Staub, 2010).

We can speculate that the difference in processing difficulty between VP3 and VP1 did not depend on WMC in our results because at VP3 condition, the facilitation has already reached a bottom asymptote (the minimum re-reading probability given the complexity of the stimuli; see Figure 2F). This lack of an effect of WMC on the facilitation might presumable be because of our relatively homogeneous pool of participants, who did not display a big enough variance in their WMC.

## 4. Experiment 2

This experiment is a replication of Experiment 1 using self-paced reading methodology. Even though eye-tracking experiments provide a more natural setting than self-paced reading, eye-tracking allows participants reading strategies that are absent in self-paced reading, such as skipping words and re-reading. Moreover, since it is possible to calculate many different eye-tracking measures, the chance of getting a false positive (a Type I error) goes up due to the multiple testing problem. Thus, one important motivation for the self-paced reading experiment was to determine whether the previous results were robust. A second motivation was to attempt a replication of the eye-tracking result using a different method. The absence of replication has been recognized as a major problem in psychology and related areas (Asendorpf et al., 2013).

### 4.1. Method

#### 4.1.1. Participants

Eighty subjects aged between 18–44 years (mean age 25 years) participated in a self-paced reading experiment in Argentina. The first 34 subjects participated in Buenos Aires and the rest in Mendoza. All participants reported to be native speakers of Spanish and were naïve to the purpose of the study. Only one participant was excluded from the analysis, since s/he reported, after the experiment had been completed, that s/he suffered from a mental disorder related to memory.

Partial-credit unit scores for the operation span test measuring WMC of the remaining 79 participants ranged between 0.373–0.882 with an average of 0.631 (*SE*: 0.015). Average character speed for the rapid automatized naming task for measuring reading skills ranged between 1.60–3.45 characters/second with an average of 2.40 (*SE*: 0.05) characters/second.

#### 4.1.2. Stimuli

The stimuli for this experiment consisted of 36 items similar to the items of Experiment 1, but with an extended spillover region. This extra region was included in case the self-paced reading task may delay the effects seen in the eye-tracking experiment.

Similarly to Experiment 1, each participant read the 36 items together with 176 unrelated sentences (120 were experimental items of three unrelated experiments and 56 sentences were filler sentences) in an individually randomized order after six practice trials; and the stimuli were presented in a Latin square design. A true-or-false comprehension task was presented after 65% of all trials in the experiment, including fillers. As in the previous experiment, the statements focused on various aspects of the stimuli, and the proportion of true and false statements was balanced.

#### 4.1.3. Procedure

Subjects were tested individually using a PC. Participants completed the three tasks at their own pace: First, they performed a rapid automatized naming task, second, an operation span task, and finally, a self-paced reading task (Just et al., 1982).

Before the self-paced reading task began, each participant was instructed to read for comprehension in a normal manner and had a practice session of six sentences. All sentences were displayed on a single line and were presented in 18 pt Arial font using Linger software (http://tedlab.mit.edu/~dr/Linger/). In order to read each word of a sentence successively in a moving window display, participants had to press the space bar; then the word seen previously was masked and the next word was shown. At the end of some of the sentences, participants had to answer whether a certain statement related to the experimental item was true or false. No feedback was given as to whether the response was correct or not. Twice during the self-paced reading task, a screen announced the number of sentences read so far and invited the participants to take a break.

#### 4.1.4. Data analysis

The appropriate transformation of the dependent variable according to the Box-Cox method (Box and Cox, 1964) was the inverse transformation. We used (−10^5^/*RT*) to improve the readability of the models (a positive *t*-value for −10^5^/*RT* corresponds to a positive *t*-value of the untransformed measure RT).

### 4.2. Results

#### 4.2.1. Comprehension accuracy

Participants answered correctly on average 77% (SE: 1) comprehension probes of all trials, and 70% (SE: 1) of the trials belonging to the experiment. The comprehension accuracy for the experimental trials ranged between 46 and 88%, while the 25th, 50th, and 75th quartiles were 62, 71, and 77% respectively. As in Experiment 1, a GLMM showed that WMC was a significant predictor of accuracy, with higher capacity leading to greater accuracy; Coef = 0.15, *SE* = 0.07, *z* = 2.02, *p* = 0.043.

#### 4.2.2. Reading times

We compared reading times at the same three regions of interest as in Experiment 1, using the same successive differences contrast coding. Since the effects appeared in the same regions as in Experiment 1, the added spillover regions were omitted from the analysis.

We removed 0.18% of the data in order to keep the residuals normally distributed; the results of the model were virtually the same without this removal.

##### 4.2.2.1. First critical region (auxiliary verb “había”)

For this region, including a quadratic term for WMC was justified according to a model comparison; an anova comparison of models based on a Chi-squared test yielded: χ^2^_3_ = 10.7, *p* = 0.013.

**Figure 3:**
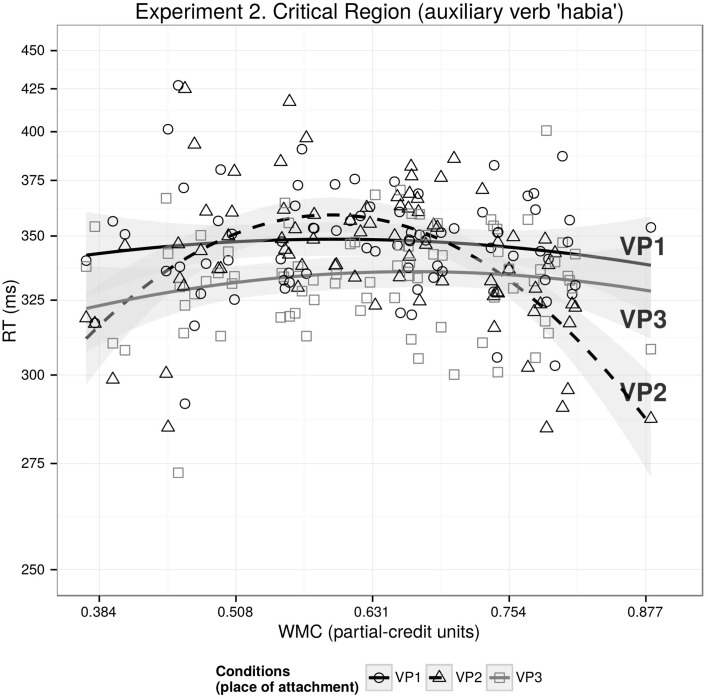
**The figure depicts the partial effects on the transformed -1/RT scale for the contributing factors condition, WMC, WMC^2^ and their interaction; random factors variance and effects due to reading skills were removed from the dependent variable using the remef function (Hohenstein and Kliegl, 2013)**.

The main results for this region are displayed in Table 3. Consistent with the indirect evidence in Experiment 1 (recall that for re-reading probabilities, we found significant facilitation in VP3 vs. VP1, but not in VP2 vs. VP1), we found a differential facilitation between VP2 and VP3: the critical region was read faster in VP3 in comparison with VP2. We also found a significant interaction between WMC^2^ and VP2-VP1 showing an inverted U-shaped effect of WMC on reading times (see Figure 3), that is, shorter reading times in VP2 vs. VP1 for low and high-WMC than for mid-WMC. In other words, speedups were seen in low as well as high-capacity readers, but not in medium-capacity readers. An interaction between WMC and VP2-VP1, even though non-significant, suggests that the speedup may be stronger for high-WMC than for low-WMC. We also found significant interactions between WMC and VP3-VP2, and between WMC^2^ and VP3-VP2. Due to these findings, we also fitted a separate model that included the VP3-VP1 contrast. This new model revealed that the effect of WMC was only relevant in relation to VP2 (as can be seen in Figure 3).

**Table 3:** **Summary of the fixed effects in the LMM with a quadratic term of WMC for reading times at first critical region in Experiment 2**.

**Predictor**	**Coef**	***SE***	***t***
VP2 vs. VP1	7.0	5.7	1.2
VP3 vs. VP2	−18.0	5.8	−3.1^*^
WMC	−2.5	6.6	−0.4
WMC^2^	−5.6	6.3	−0.9
RS	−25.6	6.6	−3.9^*^
VP2 vs. VP1 : WMC	−7.3	4.2	−1.7
VP3 vs. VP2 : WMC	9.3	4.2	2.2^*^
VP2 vs. VP1 : WMC^2^	−11.2	4.0	−2.8^*^
VP3 vs. VP2 : WMC^2^	10.3	4.0	2.6^*^
VP2 vs. VP1 : RS	6.5	4.1	1.6
VP3 vs. VP2 : RS	−9.4	4.1	−2.3^*^

* indicates a significant effect at α = *0.05*.

As expected, subjects with higher reading skills scores tended to have shorter reading times, but we also found an unpredicted interaction of reading skills with VP3-VP2 showing that as the reading skill score increases, reading times at the critical region get increasingly shorter for VP3 in comparison with VP2.

##### 4.2.2.2. Second critical region

For these regions a quadratic term for WMC was not justified, so we report the main findings for the model including only linear terms for WMC and reading skills. As in the previous region, there was a speedup for VP3 in comparison VP2, which was independent of WMC (Coef = −7.17, *SE* = 3.97, *t* = −1.81). The results showed reading skills to be significant as well: subjects with a higher score tended to have shorter reading times (Coef = −30.11, *SE* = 7.46, *t* = −4.03).

### 4.3. Discussion

The main results of the self-paced reading study are an inverted U-shaped effect of WMC on reading times for the first critical region for the condition where the extra material modified the VP (VP2) in comparison with the condition with the short dependency (VP1), and a speedup at the two critical regions when the extra material modified the VP that contained the subcategorizing verb (VP3) in comparison with when it modified the intermediate VP (VP2).

The study thus shows that individual differences associated with working memory have an impact in reading strategies for processes associated with build-up of expectations and retrieval. Moreover, this study provides more evidence for a differential effect that depends on whether the VP that contains the head of the dependency is modified, as predicted by the activation-based model, but not by DLT and the expectation account.

We found that when the extra material modifies the VP where the dependency is completed (VP3), participants showed a speedup in comparison with the condition where the extra material modifies the intermediate verb (VP2). Since the dependencies in both conditions had the same length, this experiment provides further evidence for facilitation because of preactivation of the subcategorizing verb as predicted by the activation-based account (Vasishth and Lewis, 2006; and consistent with Figure 1F).

The data also showed a surprising inverted U-shaped interaction between WMC and VP2-VP1 conditions. An analogy to exam-taking may explain how two different underlying causes may lead to a process finishing early: students leave an examination hall early either because they do not have the resources (knowledge, skills, etc.) to complete the exam (i.e., they effectively give up), or because they have the resources in excess and can complete the exam quickly. Similarly, there may be two different reasons for the shorter RTs: Low-WMC subjects may read fast because they have done a shallow parse due to not having enough computational resources (probably using a good-enough parsing heuristic see: Ferreira et al., 2002; Ferreira and Patson, 2007), while high-WMC participants may read fast because they did a complete parse and still had enough resources to take advantage of the build-up of expectations (see the right part of Figures 1E,F). Medium-WMC participants, however, may have built a complete parse but either did not have enough resources available for the build-up of predictions of the upcoming head, or the memory-driven locality effect offset the facilitation due to expectations. The difference between this study and the eye-tracking study may be due to the increased task demands of self-paced reading and the impossibility of making regressive saccades. This difference is also evident from the lower comprehension accuracy in self-paced reading in comparison with eye-tracking (70 vs. 82%).

As in the previous experiment, the speedup at the critical region depends only on WMC when the dependent-head distance is increased without a modification of the VP that contains the head (VP2-VP1), while the speedup is independent of WMC when distance is increased by a modification of the VP that contains the head (VP3-VP1). As it was shown in Figures 1B,F, it is expected that a facilitation that depends on WMC will have a bottom asymptote since the duration of the reading times cannot be zero and presumably there is a minimum time needed (for recognizing the word, pressing the space bar, etc). Since the activation-based model predicts stronger facilitation for VP3 in contrast to VP2, it also predicts that the effect of WMC on VP3 will reach the bottom asymptote earlier than on VP2 (and thus showing a “flat” WMC effect if all the participants have a relatively high WMC). It should be noted that for the extremely high values of WMC, however, the speedup of VP2 is stronger than of VP3, which is not predicted by the activation-based model (and neither by the expectation account or DLT). However, this is true for a few subjects, and it may be due to the lack of data for the extreme values of WMC.

In addition, the results showed that the facilitation due to preactivation (VP3 vs. VP2) “lasts longer.” This is in some way parallel to the findings of Experiment 1, where the facilitation at VP3 condition (this time in comparison with VP1) appeared both in a different measure (re-reading instead of first pass regression probabilities) and it spilled over to the second critical region.

## 5. General discussion

A major contribution of this paper is the finding that participants' WMC affects the processes involved in the dependency resolution. Even though recent research has shown that in some cases the relevant measure of individual difference to explain reading strategies is related to experience with language rather than memory (vocabulary size in Prat, 2011; reading speed in Traxler et al., 2012), by taking into account the results of a rapid automatized naming task, which reflects experience with language, the current study showed WMC as measured by the operation span task to be a fruitful index of individual differences (at least for dependency resolution). Even though long-distance dependency completion is widely assumed to depend on the available working memory (but see Waters and Caplan's approach to working memory: Waters and Caplan, 1996; Caplan and Waters, 1999; Waters and Caplan, 2001), this is, to our knowledge, the first study showing that WMC modulates the reading times and regressions at the head of long-distance dependencies, as predicted by both DLT (Gibson, 2000) and the activation-based model (Vasishth and Lewis, 2006). The findings are consistent with the recent work of Caplan and Waters (2013). In this work, the authors argue that working memory supports retrieval in points of high processing load, which are identified by regressive saccades and longer self-paced reading times that enable better comprehension. In addition, our results show the added value of analyses that take individual variation into account instead of averaging over the data of participants (among others: Underwood, 1975; Brown and Heathcote, 2003; Traxler et al., 2005; and more recently Kliegl et al., 2011; Traxler et al., 2012; Payne et al., 2014).

The results of Experiments 1 and 2 together suggest that increasing the distance of the dependency affects the parsing of the head of the dependency in different ways, depending on whether the intervening material modifies the upcoming head or not. As predicted by the activation-based model (Vasishth and Lewis, 2006) but not by DLT or the expectation account, the facilitation is stronger when the intervening material modifies the upcoming head even when the length of the dependency is the same.

The increase of expectation-based facilitation at the subcategorizing head depends on adding lexical material that helps to sharpen predictions on the location of the upcoming head. However, the increase of lexical material also has its cost in memory processes, so expectation-driven facilitation seems to be noticeable as a speedup or as the decrease of regressions for participants with enough resources to overcome the difficulties caused by adding the extra lexical material (at least when the added facilitation due to the preactivation of the subcategorizing VP is absent). This predicts a monotonic effect of WMC, namely, when distance is increased, the difficulty for low-WMC is reduced as WMC increases, which turns into facilitation for high-WMC. While that was the case for our eye-tracking study (Experiment 1), this interaction was more complex than predicted for the self-paced reading task (Experiment 2).

Expectation-driven facilitation reduced the probability of regressions depending on the WMC of the participants of our eye-tracking study (Experiment 1), so that locality effects decreased as WMC increased until they became increasing antilocality effects. However, for the participants of the self-paced reading task (Experiment 2), the effect of WMC had an inverted-U shape, showing speedups in comparison with the short dependency condition for both high- and low-capacity readers. Since WMC predicted better comprehension accuracy, we assume that there are different underlying processes behind these two speedups, and only high-WMC readers are assumed to speed up because their WMC allowed them to parse the sentence and predict the upcoming lexical material. Since locality effects are assumed to be a response to either a memory overload (Gibson, 1998), the use of more computational resources (Gibson, 2000), or higher retrieval costs (Vasishth and Lewis, 2006), theories that predict locality effects would not predict that low-WMC participants would speed up in comparison with mid-WMC readers when the distance between head and argument is increased. In fact, there is ample evidence that proposes that individual differences in WMC reflect limitations in attention allocation for goals, especially in the face of interference or distraction (for a review see Engle, 2002).

There is independent evidence that high working memory load may lead to faster processing; this comes from the self-paced reading studies of Van Dyke and McElree (2006), who found that when subjects were presented with a memory load (a series of words to recall later) prior to reading a sentence, reading times were shorter and comprehension accuracy was lower in comparison with the conditions without the memory load. It seems that when the comprehender is parsing material while being engaged in processes that tax memory, a possible reading strategy is to disengage from the memory load sooner by reading faster. These results are in line with good-enough parsing (Ferreira et al., 2002; for a review: Ferreira and Patson, 2007), which states that the parser is not necessarily trying to achieve a fully specified representation of the sentence and that it might accept a partial or inconsistent representation. Furthermore, the findings converge with studies showing that low-WMC subjects may take less time when ambiguities are present (but they had worst accuracy) than high-WMCs (MacDonald et al., 1992; Pearlmutter and MacDonald, 1995; von der Malsburg and Vasishth, 2013), that they can read superficially enough to draw contradicting conclusions from a text (Oberauer et al., 2006); and that older adults' increase their reliance on heuristic-like good-enough processing to compensate for age-related deficits in WMC (Christianson et al., 2006).

Since this speedup for low-WMC readers is hypothesized to be a response to an incomplete parse of the more memory demanding condition, the speedup should appear together with a trade-off in the accuracy of the dependency completion. However, the true-or-false statements used for testing the participants' comprehension accuracy included many aspects of the stimuli in order to verify that they paid attention to the sentences, but they did not target exclusively whether the dependency was understood. Participants could in principle know whether the statement after the stimulus sentence was true or false, even without a complete understanding of the previous probe sentence. In addition, they could answer wrongly because they misunderstood other aspects of the sentences. The reason for this shortcoming is twofold: First, since most of the previous studies on locality effects examined only on RTs (except for McElree et al., 2003), the design of the experiment was not meant to explore the comprehension accuracy. Second, the nature of the stimuli made it almost impossible to make short comprehension questions that could test only the dependency; this is so because the comprehension questions would ideally need to test whether sentences such as “it was commented that someone had fired who” are correct. Even though neglecting a deeper analysis of the sentence comprehension task is the normal state of affairs in psycholinguistics, it is a long-standing shortcoming in psycholinguistic research (but see: Christianson et al., 2001; Ferreira et al., 2001).

In sum, we have presented evidence that locality/antilocality effects are modulated by the participants' WMC. However, the exact relationship between WMC and expectations remains elusive. Two possible explanations are: (i) the prediction processes benefit from more WMC being available, as illustrated by Figure 1D, such that high-capacity readers may have a more precise expectation of the upcoming material, or they may be able to maintain the predictions for a head generated by the displaced argument (the wh-element in the experiments) for a longer time; or (ii) the prediction processes by themselves are unaffected by WMC (Figure 1C), while the stronger facilitation for high-WMC takes place due to the prediction processes being less affected by memory-driven locality effects.

### Conflict of interest statement

The authors declare that the research was conducted in the absence of any commercial or financial relationships that could be construed as a potential conflict of interest.
